# Diabetic Cheiroarthropathy: A Recognised but Forgotten Complication—A Cross-Sectional Study Among Type 2 Diabetes Mellitus Patients in Seremban, Malaysia

**DOI:** 10.21315/mjms-10-2025-681

**Published:** 2026-04-30

**Authors:** Zairul Nizam Z Fithri, Justin Vijay Gnanou, Shevaaliiny Ravi Chandran, Shobna Sai Letchumenan, Mohammad Arshad Ikram

**Affiliations:** 1 Orthopaedic Unit, Department of Surgery, School of Medicine, IMU University Clinical Campus, Seremban, Negeri Sembilan, Malaysia; 2 Department of Human Biology, School of Medicine, IMU University, Kuala Lumpur, Malaysia; 3 School of Medicine, IMU University Clinical Campus, Seremban, Negeri Sembilan, Malaysia

**Keywords:** disabilities of arm, shoulder and hand score, cheiroarthropathy, limited joint mobility syndrome, prevalence, upper limb dysfunction

## Abstract

**Background:**

Diabetic cheiroarthropathy (DCA), or limited joint mobility, is a common yet underdiagnosed complication of diabetes, causing painless hand stiffness and reduced arm function, and local Malaysian data about this condition are scarce. This study aimed to ascertain DCA prevalence among type 2 diabetes mellitus (T2DM) patients in Seremban, Malaysia, and to explore its effects on their daily function.

**Methods:**

A prospective, cross-sectional study was conducted from September 2024 to August 2025 at a primary healthcare clinic with 320 adults with T2DM. Patients with pre-existing hand problems were excluded. DCA was diagnosed using prayer and tabletop signs. The range of wrist movement was measured with a goniometer, and upper limb function was determined using the QuickDASH questionnaire. The data were analysed using logistic regression to find links between DCA and demographic and clinical characteristics.

**Results:**

Out of 320 patients, 43.8% had DCA. In the univariate analysis, older age and longer diabetes mellitus (DM) duration were linked to having DCA. In the multivariate regression, smoking and DM duration were better predictors of DCA. Compared to patients without DCA, those with DCA had a smaller range of wrist movement (71.9° vs. 75.9°) and higher QuickDASH scores (7.2 vs. 4.5), indicating a greater severity of disability.

**Conclusion:**

DCA is a prevalent and clinically significant complication among T2DM patients in Malaysia, contributing to functional impairment. Longer DM duration and smoking may increase the risk of DCA. Regular screening using simple tests such as the prayer sign can help with the early detection and prevention of musculoskeletal complications among T2DM patients.

## Introduction

Diabetes mellitus (DM) complications present with myriad musculoskeletal manifestations, including limited joint mobility syndrome, otherwise known as diabetic cheiroarthropathy (DCA) (1). DCA constitutes part of a triad syndrome that commonly occurs with adhesive capsulitis and restricted hip motion (2). Patients present with ulnar to radial progression of limited extension of the interphalangeal and metacarpophalangeal joints (3). DCA is the result of noninflammatory thickening and progressive painless stiffness of the joints and presents with tight, waxy skin of hands and fingers (1). It is related to the nonenzymatic accumulation of advanced glycation end-products (AGEs) that cause excessive crosslinking of the skin and tendons (4).

DCA can be diagnosed using simple clinical tests such as the prayer and tabletop signs (5). Failure to completely flatten one’s palms together, as if in prayer, indicates contractures in the metacarpophalangeal, proximal interphalangeal, and distal interphalangeal joints. Similarly, observing the hand’s capacity to flatten on a table’s surface whilst standing supports the identification of contractures in the metacarpophalangeal joints (4).

However, DCA is commonly overlooked as a significant complication of DM. It is overshadowed by ophthalmologic, renal, and cardiovascular events, despite being frequently associated with macro- and microangiopathic derangement (4, 6), which accounts for up to a fourfold risk of neuropathy, retinopathy, and nephropathy (7). When coupled with peripheral neuropathy, DCA can significantly limit one’s daily activities, especially self-care (1).

There is a clear gap regarding the awareness of this complication among medical professionals (8), and it is absent in the Malaysian diabetes management guidelines (9). A literature search using the keywords “diabetic cheiroarthropathy” and “limited joint mobility syndrome” revealed no relevant studies in Malaysia. Given the scarcity of local evidence, this study assessed the prevalence of this complication in our Seremban population and evaluated DCA as an indicator of upper limb dysfunction measured using the abbreviated disabilities of the arm, shoulder, and hand (QuickDASH) questionnaire (10). To the best of our knowledge, our study is the first to evaluate the prevalence of DCA in Seremban, Malaysia.

## Methods

This prospective, cross-sectional observational study was carried out between September 2024 and August 2025. The consecutive sampling method was used to select patients during routine diabetic clinic follow-ups in Klinik Kesihatan Seremban. To minimise selection bias, patients’ symptoms and functional complaints were not specifically screened prior to the study. Based on a reported DCA incidence of 21.7% (11) with a precision of +5%, a sample size of 262 patients was calculated.

Patients were included if they were Malaysian adults with physician-confirmed type 2 diabetes mellitus (T2DM). Patients with pre-existing hand disorders (e.g., amputations and active ulcers, rheumatoid arthritis, scleroderma, Dupuytren’s, and hand osteoarthritis) or deformities of the hand caused by trauma or surgery, including existing contractures, were excluded. Pregnant patients and individuals with liver or neurological disorders were also excluded.

Recruited patients underwent clinical assessment for DCA using the prayer and tabletop signs (Figure 1). These tests are established clinical methods of identifying DCA and are easily administered and reproducible; they also have the benefit of being binary (yes/no) rather than graded assessments, thus limiting interobserver variability. DCA was designated for any joint of both hands that failed to make contact. As patients performed these clinical tests, the range of motion of their wrist joint in extension was measured using a standard goniometer (Seca, GmbH, Germany). The average of three measurements was recorded as the patient’s range of motion. Thereafter, a validated 11-item QuickDASH questionnaire was administered to assess upper limb function (10). Validated Malay, simplified Chinese and Tamil translations of the QuickDASH questionnaire from the original English version were available for patients (obtained and downloaded from https://dash.iwh.on.ca/available-translations). Ranging from 0 to 100, a high QuickDASH score indicated a higher level of upper limb dysfunction. Patients’ medical records were reviewed for general demographic data, DM duration, smoking status, body mass index (BMI), and blood glucose control based on the latest glycated haemoglobin (HbA1c) level.

All data were analysed using Statistical Package for the Social Sciences (SPSS) version 28 (IBM Corp., Armonk, NY, US) to determine the overall prevalence of DCA. Univariable logistic regression analysis was conducted for binary outcomes vs. continuous variables, and the chi-square test (*x*^2^) was performed for binary vs. categorical variables. All data were assessed for completeness and relevance prior to analysis. Any missing or incomplete medical data were excluded. Multivariable logistic regression of all clinically relevant variables, including significant results on univariable analysis, expressed as adjusted odds ratio (OR) with 95% confidence interval (CI), was analysed for possible associations between variables collected.

## Results

All patients were recruited from a community health centre when attending diabetic follow-up at their noncommunicable disease clinic. A total of 320 patients were recruited; their ages ranged from 29 to 87 years, with an average of 64.30 ± 10.98 years. Slightly more than half of the participants (57.5%) were male. The prevalence of DCA was 43.75%.

Age showed a statistically significant but weak positive association with DCA; patients with DCA tended to be older (*P* = 0.002). There was no significant relationship between patient gender and DCA (*P* = 0.362). Participants with DCA tended to have longer DM duration (*P* = 0.005). Patients without DCA had a slightly higher BMI compared to those with DCA, but this difference was statistically insignificant (*P* = 0.083). Smoking status did not have a statistically significant association with DCA (*P* = 0.09). Blood glucose control based on HbA1c levels also showed no significant difference in HbA1c levels between patients with and without DCA (*P* = 0.492). These findings are summarised in Table 1.

### Univariable Analysis of Parameters

To study the association of these variables with DCA, univariable analyses were performed. Patient age and DM duration were significantly associated with DCA (OR = 1.03/year, *P* = 0.004; OR = 1.05/year, *P* = 0.005, respectively). BMI was inversely associated with DCA (OR = 0.94/kgm^−2^ increase, *P* = 0.015). Gender, smoking, and HbA1c levels were not significantly associated with DCA (*P* > 0.05 for all variables). This is shown in Table 2.

### Multivariable Analysis of Parameters

To identify the probable predictive value of age, gender, DM duration, BMI, smoking status, and HbA1c level (glucose control), a multivariable logistic regression model was used. Age, DM duration, and BMI showed statistically significant associations in univariate analysis, while gender, smoking status, and HbA1c were chosen as clinically relevant variables. The multivariable logistic regression model demonstrated adequate calibration (Hosmer–Lemeshow *χ*^2^ = 5.46, *df* = 8, *P* = 0.707). The model explained 7.1% of the variance in DCA presence (Nagelkerke *R*^2^ = 0.071) and achieved an overall classification accuracy of 57.7%. The area under the Receiver Operating Characteristic (ROC) curve was 0.629, indicating fair discrimination ability. DM duration (OR = 1.035; *P* = 0.059) and smoking status (OR = 2.240; *P* = 0.079) had borderline predictive values. This implied that longer DM duration slightly increases the odds (~3.50% per year) of developing DCA, while smokers have ~2.24 times higher odds of developing DCA. Variables such as age, gender, BMI, and HbA1c level (glucose control) were not independently predictive of DCA after adjustment.

### Association Between DCA and Wrist Range of Motion

The average wrist extension for all patients was 74.1° ± 6.6°. Patients with DCA had significantly less range of motion compared to those without (*P* < 0.001; Table 3). Regression analysis revealed a statistically significant negative correlation between range of motion and the presence of DCA (B = −0.101; OR = 0.904; 95% CI: 0.870, 0.939; *P* < 0.001), indicating that greater range of wrist extension was associated with reduced odds of DCA.

### QuickDASH Score as a Consequence of DCA

Finally, the QuickDASH score versus the presence of DCA was analysed. The mean QuickDASH score for the entire cohort was 8.93 ± 11.35 (range: 0.00–72.73). As can be seen from the box plot (Figure 2), the median QuickDASH score for patients with and without DCA was 7.2 and 4.5, respectively. This suggests that, on average, patients with DCA reported slightly more severe and variable disability with a wider interquartile range. Overall, patients who had DCA had significantly worse upper limb dysfunction compared to those without DCA (*P* = 0.002).

## Discussion

As a type of limb dysfunction, DCA is a condition that is often overshadowed by other diabetic complications such as renal failure, diabetic retinopathy, and cardiovascular issues. However, this condition may readily worsen and cause problems with patients’ use of their limbs, leading to limitations in daily activities (including medication consumption) and even psychological distress (9, 12, 13).

The results indicated that a relatively high percentage of patients (43.75%) had DCA. This study exclusively investigated individuals with T2DM; expansion of the catchment to involve type I patients could potentially increase this number. The prevalence rate falls on the upper limits (90th percentile) of published data of 3.5% to 58.0% for all types of DM (1) and is higher than rates reported by some authors specifically for T2DM. Paul and Gnanamoorthy (4) reported a prevalence of 18.3% among 251 T2diabetic patients, while Asmath et al. (11) diagnosed DCA in 21.7% of 300 patients. Arkkila et al. (6) found the prevalence of DCA among their patients (average age 61.3 years) to be even higher at 60%. Our population data puts DCA prevalence within these ranges. This might be a reflection of the large number of older patients (average: 64.30 ± 10.98 years) presenting for follow-up in the current study. It has been postulated that reports with lower DCA prevalence tended to have younger patients with shorter DM duration (6, 14). Based on the range and average ages of the populations of the cited papers and our own study, that may be the case.

The univariable analysis showed that age and DM duration had positive independent associations. Other reports have shown a similar positive link (11, 15). Paul et al. (16) found a significant correlation and higher risk between DCA and DM duration, suggesting that prolonged DM duration plays a role in excessive nonenzymatic AGE buildup. Examining 400 patients, Pandey et al. (7) found a 40.5% prevalence of DCA with a stronger correlation with DM duration of more than 10 years. However, using multivariate regression analysis, the present study found only DM duration and smoking to be better predictors of DCA. While the former has been noted as a predictor of DCA in other studies, evidence of the effect of smoking on DCA is not as extensive. Although it is controversial in terms of diabetic retinopathy progression among patients with T2DM (17, 18), smoking, among other factors such as hyperglycaemia, has nonetheless been implicated in the rapid progression of renal and nerve complications through inflammation and oxidative stress (19, 20). Clair et al. (21) further postulated that smoking might have direct toxic effects through tissue hypoxaemia and microvascular insufficiency. The resulting microvascular complications of smoking might explain the present study’s results (22). Arkkila et al. (6), on the other hand, found no DCA association with smoking in their patient group. This lack of robust evidence deserves further in-depth investigation. The findings in the present study only show a borderline but still significant association. Prolonged DM duration has repeatedly been associated with DCA incidence. This study suspects that smoking is primarily a case of insufficient power to detect greater significance. An older study by Eadington (23) showed similar associations between limited joint mobility, smoking and DM duration; although the patient population was type 1 DM, the interpretation can be extrapolated to T2DM (24). It appears that smoking cumulatively worsens the risk of developing DCA, particularly in those with longer DM duration. This underscores the importance of smoking cessation among patients with diabetes.

The univariate analysis also indicated that BMI had a slight independent negative correlation with DCA. However, it was not predictive after adjustment in the multivariate regression analysis. This apparently contradictory inverse relationship might be due to several reasons. BMI is a crude proxy for the overall body composition; a lower BMI might be due to muscle mass deterioration or sarcopenic obesity associated with poorer glycaemic control and cumulative microvascular damage, which could predispose one to musculoskeletal complications, including DCA (23). Since DCA is related to prolonged hyperglycaemia and nonenzymatic AGE accumulation and eventual collagen crosslinking (4), the higher occurrence of DCA seen could also reflect an overall poorer hyperglycaemic control among the cohort, since the control level determined using HbA1c is just at a particular point in time rather than the control in its entirety. Additionally, the present study’s data indicated a weak but statistically significant association between BMI and DM duration (ρ = −0.18; *P* < 0.05). It is possible that the cohort of DCA patients with lower BMI had DM longer than their counterparts with higher BMI. The association between DCA and BMI is tenuous; some researchers have found no association (6, 7, 25) while others have (1). Hill et al. (26) wrote about BMI being linked to tendinopathy in diabetics but not with DCA. Further investigation involving detailed anthropometric examination to assess body fat and muscle mass changes should provide more relevant insights.

Reports indicate an association between DCA and raised HbA1c levels (7, 14), although the current study’s findings do not reflect this. Singh et al. (27) reported worse diabetic control in patients with rheumatological hand manifestations, but did not elaborate on what those manifestations were. Prolonged hyperglycaemia is the main driver of protein glycation and AGE formation (16) even though a positive association with DCA is not universal (4, 26, 28). One possibility is that the onset of glycation irreversibly persists from long-term cumulative damage despite eventual glycaemic control (26). Previous research has pointed towards the association between DCA and atherosclerotic vascular disease (which includes cerebrovascular and cardiovascular events) as well. It is therefore judicious to identify patients who have DCA and monitor them closely for these life-threatening conditions. Some authors went further to suggest that DCA may be useful for identifying patients at risk of developing foot problems after demonstrating a correlation between both (5, 25, 29). Recently, it has been suggested that DCA might indicate susceptibility to hyperglycaemic multiorgan fibrosis (28). This new revelation requires further, more exhaustive validation with radiological modalities and circulating biomarkers such as AGEs.

Another factor that has been studied in patients with DCA is wrist range of motion. A few studies have evaluated composite hand motions and verified the reduced range of motion in patients with diabetes (15, 30). Similar to other studies (2, 28), the present study’s findings of limited wrist extension accompanying DCA appear to show it as a local independent risk factor, a similar finding to others. This suggests its usefulness as a further indicator of the presence or onset of DCA. Due to the limited studies available on this issue, more extensive research involving a sizeable population needs to be conducted in view of the possible implications for self-care and daily life activities.

This study also included QuickDASH as a quick way to assess upper limb function. Based on the data, there was a wide variation among the patients’ QuickDASH scores. While the average score was relatively low (8.93 ± 11.35), the large standard deviation indicated a wide variation in upper limb dysfunction among the patients. In this study, patients with DCA had worse upper limb dysfunction compared to those without DCA. Joshi et al. (31) also examined upper limb dysfunction using the DASH questionnaire and noted a score of 10.3 ± 11.9, with an SD similar to the present study. Gokcen et al. (32) further divided their patients with DCA according to diabetes type and found that DASH scores were worse for patients with prediabetes or type 1 DM. Other authors have previously investigated the relationship between upper limb dysfunction and DM using various other outcome measures. Shah et al. (33) used the Shoulder Pain and Disability Index and found that 31% of their patients with diabetes reported substantial upper limb pain and disability.

DCA identification has value for preventing not only upper limb dysfunction but also other microangiopathic complications. Healthcare systems in countries such as India have advocated for greater awareness of DCA, but it is still commonly overlooked. Its diagnosis is simple, and management may be initiated when detected early (28, 32, 34), before lasting functional complications develop, begin to affect one’s quality of life and require specific and individualised orthopaedic interventions (3).

The present study found some areas requiring additional work. Principal among them is a more robust nationwide epidemiological study examining demographic, social, and phenotypic parameters, including the presence of microvascular complications that serve as confounding factors. The study recommends forwarding initiatives to raise DCA awareness and implement systematic screening as part of routine clinical practice.

### Limitations

The authors acknowledge several limitations of this study. First, being a cross-sectional study among patients with documented T2DM limits the causal inferences that can be made. In addition, the population could not be verified as representative of the entire nation of Malaysia. Furthermore, we a more health-conscious and/or more symptomatic subgroup of the overall population may have been sampled, thus inadvertently introducing selection bias despite the authors’ best efforts.

Second, the clinical determination of DCA lacked quantitative objective measurement and may be regarded as a limitation. The study used wrist extension alone as a determination of movement reduction purely for convenience during the performance of other clinical tests in a busy setting. Collation with other wrist movements may have strengthened the findings.

Third, diabetic microangiopathic complications that may independently influence upper limb dysfunction were specifically evaluated in this study. However, the choice of variables such as DM duration and HbA1c levels as covariates served as indirect markers of the microvascular burden but did not eliminate confounding entirely. Although minimal in number, some missing data that were excluded from the analyses may have residual statistical power.

Finally, although validated and appropriate for an extremely busy outpatient environment, the QuickDASH questionnaire may have been too simplistic and could probably be supplemented with other related disease outcome measures, such as the SF-36 quality of life questionnaire.

## Conclusion

Though it is a significant complication of DM, DCA is an often-overlooked condition. A large percentage of the examined T2DM population did, in fact, have DCA, which translated into upper limb dysfunction based on a lower QuickDASH score. Multivariate analysis showed that DM duration and smoking were predictors of DCA in the population. Limited wrist extension, as measured by the prayer and tabletop signs and wrist range of motion, is frequently observed in conjunction with DCA and might be a useful indicator of the condition. The paucity of local studies on this underemphasised condition should prompt more extensive research into this complication. Future studies incorporating other microangiopathic complications, such as retinopathy and nephropathy, and involving multiple sites reaching various communities should provide a more detailed picture of DCA among Malaysia’s diabetic population.

## Figures and Tables

**Figure 1:**
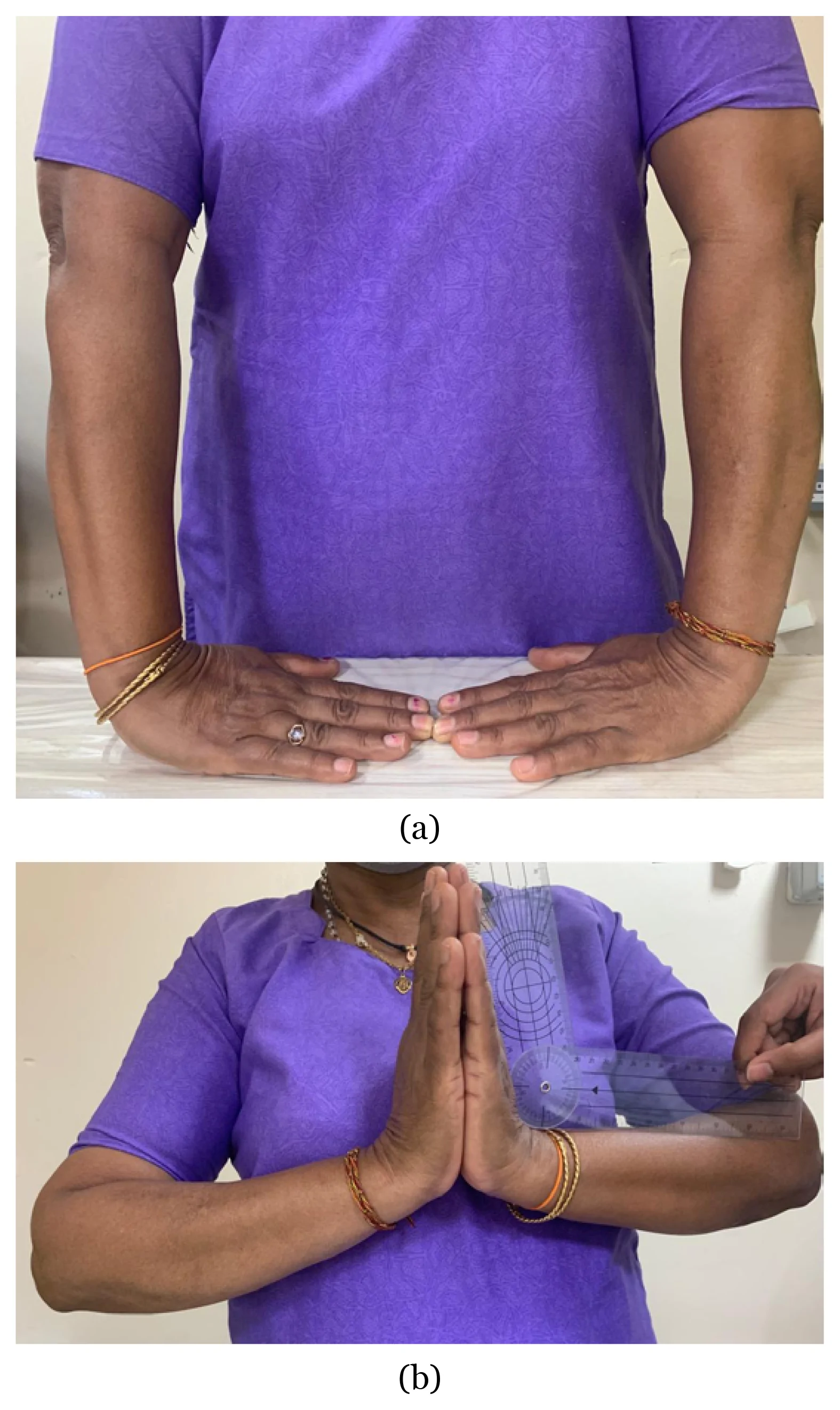
Clinical tests for DCA: (a) demonstration of the tabletop sign and (b) demonstration of the prayer sign with wrist extension measurement using a goniometer

**Figure 2:**
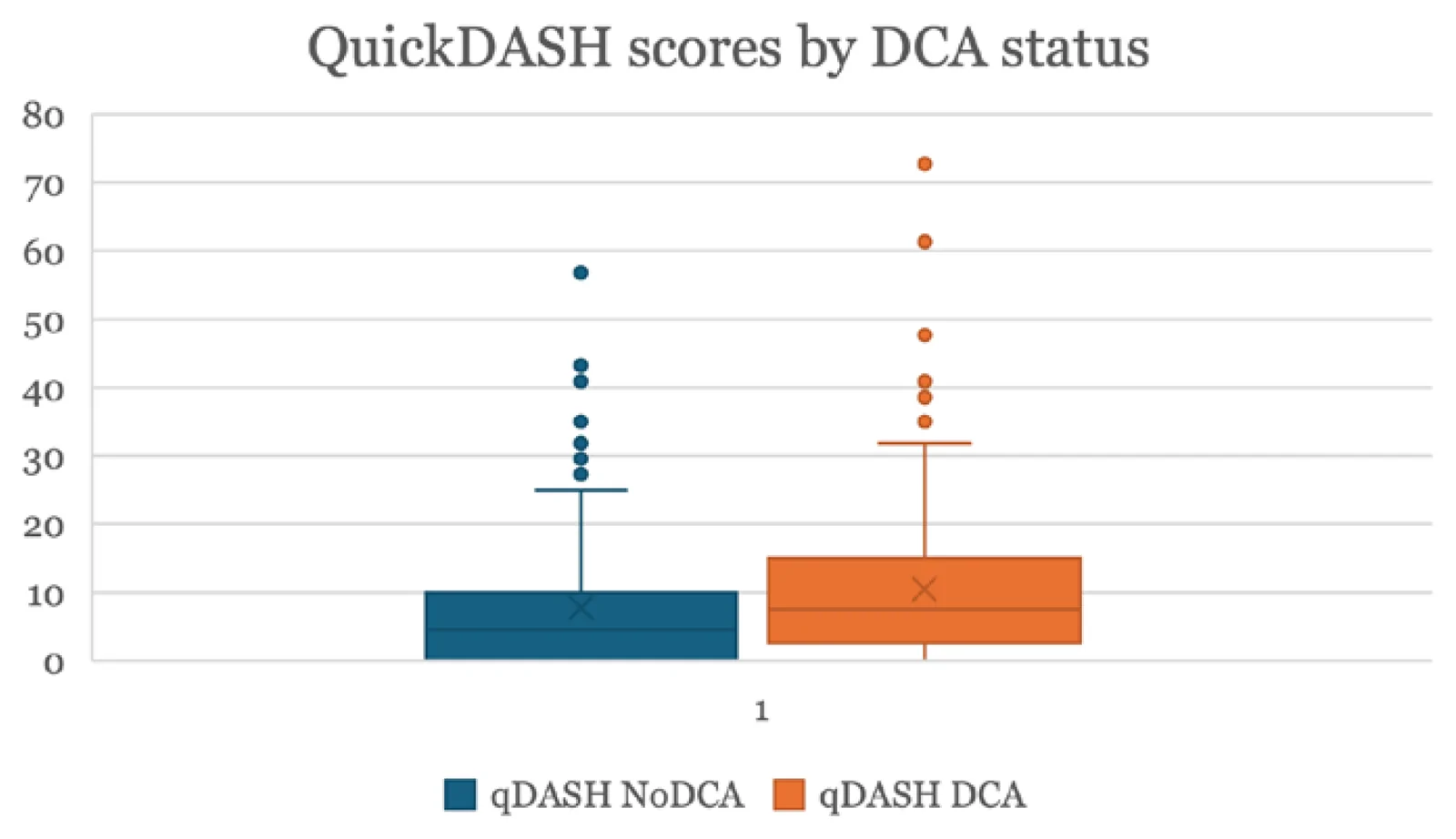
QuickDASH score by DCA status

**Table 1:** Demographic and clinical characteristics of patients by DCA status

Variable	No DCA (*n* = 180)	With DCA (*n* = 140)	*P*-value
Age (years)	62.7 ± 12.2	66.3 ± 8.7	**0.002**
Male	108 (60.0%)	76 (54.3%)	0.362
Female	72 (40.0%)	64 (45.7%)	
Duration of DM (years)	9.7 ± 7.1	12.1 ± 7.2	**0.005**
BMI (kg/m^2^)	27.7 ± 5.4	26.2 ± 4.7	0.083
Smoking (%)	6.1	12.1	0.090
HbA1c (%)	7.5 ± 1.7	7.4 ± 1.6	0.492

**Table 2:** Univariable analyses of variable association with DCA

Variable	OR	95% CI	*P*-value
Age (per year)	1.03	1.01, 1.05	**0.004**
Gender (male vs. female)	1.26	0.81, 1.97	0.305
Duration of DM (per year)	1.05	1.01, 1.08	**0.005**
BMI (per kg/m^2^)	0.94	0.09, 0.99	**0.015**
Smoking	2.07	0.89, 4.81	0.090
HbA1c (per %)	0.95	0.83, 1.09	0.492
